# Clinical Spectrum and Management of Paediatric Chronic Pancreatitis

**DOI:** 10.7759/cureus.102465

**Published:** 2026-01-28

**Authors:** Nida Zeeshan, Muhammad Junaid, Muhammad Umer Khaqan, Faisal Dar, Iqtadar Seerat

**Affiliations:** 1 Paediatric Gastroenterology/Hepatology and Child Health, Pakistan Kidney and Liver Institute and Research Centre, Lahore, PAK; 2 Hepatopancreatobiliary and Liver Transplant Surgery, Pakistan Kidney and Liver Institute and Research Centre, Lahore, PAK

**Keywords:** chronic pancreatitis, epigastric pain, frey’s procedure, lipase level, paediatric patients

## Abstract

Objective

Chronic pancreatitis is a long-standing inflammatory condition of the pancreas that leads to progressive damage and impaired pancreatic function, resulting in various complications. Although historically considered uncommon in children, its incidence is increasing. This study aimed to describe the clinical spectrum, diagnostic findings, and management approaches of paediatric chronic pancreatitis and the importance of multidisciplinary team management to optimise patients in a tertiary care setting.

Method

This retrospective observational cohort study was conducted in the Department of Paediatric Gastroenterology and Hepatology at the Pakistan Kidney and Liver Institute and Research Centre (PKLI&RC), Lahore. Medical records of children diagnosed with chronic pancreatitis between September 2021 and September 2023 were reviewed. Demographic characteristics, clinical presentation, laboratory findings, imaging results, and treatment modalities were analysed using SPSS (IBM SPSS Statistics for Windows, IBM Corp., Armonk, NY).

Results

A total of 23 children met the inclusion criteria; two were lost to follow-up, leaving 21 patients for analysis. There were 11 males and 10 females, and the mean age of the children was 10.52 years. In terms of residence, the largest proportion (57.2%) resided in Punjab. Epigastric pain was reported in all patients. Parental consanguinity was present in 85.7% of cases, and the aetiology was idiopathic in 71.4%. Pancreatic amylase was elevated in the majority of patients (90.5%), and lipase levels were raised in 81.0% of cases. The CT scan findings varied, with 38.1% of patients exhibiting a moderately dilated pancreatic duct. Magnetic resonance cholangiopancreatography (MRCP) findings revealed minimal prominence of the pancreatic duct in 42.9% of cases. Endoscopic retrograde cholangiopancreatography-guided stent placement was required in 23.8% of patients. Medical management was the initial treatment approach, while 47.6% of patients required surgical intervention for persistent or refractory symptoms.

Conclusion

Early diagnosis of paediatric chronic pancreatitis by clinical assessment, biochemical and genetic testing, and radiological evaluation is essential for effective management. While medical therapy is the keystone of initial treatment, endoscopic and surgical interventions play an important role in selected patients with refractory symptoms. A tailored, multidisciplinary approach is especially crucial in resource-limited settings to improve patient outcomes.

## Introduction

Pancreatitis was previously considered uncommon in children compared to adults; however, recent studies have demonstrated a significant increase in its incidence worldwide. A study by Liu et al. reported a rising global burden of pancreatitis among children and adolescents [1]. Despite this trend, the incidence of both acute pancreatitis (AP) and chronic pancreatitis (CP) in Pakistani children remains largely unknown.

CP is an irreversible inflammatory disorder that leads to continuous, progressive changes in the pancreas, including loss of acinar cells, fibrosis, ductal alterations, and calcifications. The diagnosis of paediatric CP in this study was based on the British Society of Paediatric Gastroenterology, Hepatology and Nutrition (BSPGHAN) guidelines [2]. The most common clinical manifestations include pain, nausea, and vomiting. Ongoing pancreatic injury may result in complications such as pseudocyst formation, as well as endocrine and exocrine pancreatic insufficiency.

Advances in diagnostic facilities have enabled earlier detection of disease and its underlying cause. These include biochemical tests (serum amylase, serum lipase, calcium level, phosphorus level, lipid profile, HbA1c, and sweat chloride level) and genetic evaluation. A study conducted by Yu et al. emphasised the importance of genetic testing for diagnostic accuracy and guiding management, particularly in children with idiopathic CP [3].

Radiological investigations, including abdominal ultrasound, computed tomography (CT), endoscopic ultrasound (EUS), magnetic resonance cholangiopancreatography (MRCP), and endoscopic retrograde cholangiopancreatography (ERCP), have significantly enhanced the assessment of anatomical changes in the pancreaticobiliary system, providing crucial insights into disease characterisation and progression [4].

Pain is a prominent presenting complaint and a significant source of distress for patients, often leading to significant impairment in quality of life. Initial management focuses on medical therapy, including opioid and non-opioid analgesics. However, in cases of refractory pain or disease complications, surgical interventions such as the Puestow procedure, Roux-en-Y pancreaticojejunostomy, Frey’s procedure, Beger procedure, Whipple procedure, or total pancreatectomy with islet autotransplantation may be required [5].

Given the limited data on paediatric CP from Pakistan, this study aims to describe the clinical spectrum, diagnostic findings, and management strategies in affected children, while highlighting the importance of a multidisciplinary approach in optimising patient outcomes.

## Materials and methods

This retrospective observational cohort study was conducted in the Department of Paediatric Gastroenterology and Hepatology at the Pakistan Kidney and Liver Institute and Research Centre (PKLI&RC), Lahore. The study duration was from September 2021 to September 2023. Paediatric patients presenting with CP during this period were evaluated.

Children up to 15 years of age diagnosed with CP were included in the study. The diagnosis of paediatric CP in this study was based on the BSPGHAN guidelines, which require the presence of at least one of the following criteria: abdominal pain consistent with a pancreatic origin accompanied by imaging findings suggestive of CP; evidence of pancreatic exocrine insufficiency with supportive imaging findings; evidence of pancreatic endocrine insufficiency with imaging suggestive of CP; or pancreatic histopathology consistent with CP [1]. All required elements were not possible in patients due to age-related challenges, limited availability of certain investigations, and resource constraints.

Data were collected using a structured proforma that included demographic variables (age, gender, residence, consanguinity, height, and weight), clinical features (epigastric pain, nausea, vomiting, fever, abdominal distention, and steatorrhoea), and aetiological factors (idiopathic causes, gallstones, anatomical malformations, genetic causes, hyperlipidaemia, cystic fibrosis, and drug-induced pancreatitis). Biochemical investigations included serum calcium, pancreatic amylase, lipase, lipid profile, HbA1c, sweat chloride testing, and autoimmune markers where indicated. Radiological evaluation consisted of abdominal ultrasound, CT, MRCP, and ERCP. Genetic testing was performed in selected patients based on availability.

Management strategies, including medical, endoscopic, and surgical interventions, were planned through a multidisciplinary team approach involving paediatric gastroenterologists, hepatopancreatobiliary surgeons, radiologists, and nutritionists. Post-treatment outcomes were assessed through telephone follow-up conducted at six weeks and three months after initiation of therapy.

Ethical approval for the study was obtained from the Institutional Review Board (IRB) of the Pakistan Kidney and Liver Institute (IRB No. 00062025). Written informed consent was obtained from the parents or legal guardians of all participating patients.

Descriptive statistical analysis was performed using SPSS (IBM SPSS Statistics for Windows, IBM Corp., Armonk, NY) software. Continuous variables were summarised as means, while categorical variables were expressed as frequencies and percentages.

## Results

During this study period, a total of 23 children met the inclusion criteria. Two were lost to follow-up, leaving 21 patients for analysis. There were 11 males (52.4%) and 10 females (47.6%). The mean age of children was 10.52 years. The majority of patients (57.2%) were residents of Punjab. Parental consanguinity was reported in 18 patients (85.7%). Epigastric pain was the most common presenting symptom and was reported in all patients. Vomiting was the next most common symptom, affecting 11 patients (52.4%), followed by nausea and steatorrhoea. The most common aetiology was idiopathic, i.e., the cause was not known, in 15 patients (71.4%). Other causes include anatomic malformation, including pancreatic divisum, which were identified in three patients (14.3%). Gallstones were identified in one patient (4.8%). Only two patients (9.5%) who underwent genetic testing were found to have a genetic cause (Table 1).

**Table 1:** Demographic and clinical characteristics of children with pancreatic disorders (n = 21)

Characteristics		n (%)
Gender	Male	11 (52.4%)
	Female	10 (47.6%)
Age (years)	2-5	1 (4.8%)
	6-12	14 (66.7%)
	13-15	6 (28.6%)
Residence	Punjab	14 (66.7%)
	Khyber Pakhtunkhwa	4 (19.2%)
	Azad Kashmir	2 (9.6%)
	Gilgit Baltistan	1 (4.8%)
Consanguinity	Yes	18 (85.7%)
	No	3 (14.3%)
Presenting complaints and clinical features	Epigastric pain	21 (100.0%)
	Vomiting	11 (52.4%)
	Nausea	3 (14.3%)
	Steatorrhoea	3 (14.3%)
	Fever	2 (9.5%)
Causes	Idiopathic	15 (71.4%)
	Anatomic malformation	3 (14.3%)
	Genetic causes	2 (9.5%)
	Gallstones	1 (4.8%)

Baseline height and weight percentiles were assessed at diagnosis. Most patients were below the 0.1st percentile for height and weight, indicating significant growth impairment at presentation (Table 2).

**Table 2:** Height and weight percentiles at diagnosis by age and gender

Age (Female)	Height Percentile	Weight Percentile
6.5 years	0.3	3.5
8 years	80	98
8.5 years	7	30
10 years	10	1.5
11 years	98	70
11 years	4	11
12 years	1	0.1
12 years	10	0.1
14 years	5	70
14 years	0.1	0.1
Age (Male)	Height Percentile	Weight Percentile
3 years	95	64
6 years	0.1	0.1
8 years	62	6.5
9 years	7	18
9 years	27	18
11 years	84	93
12 years	0.1	0.1
13 years	76	12
14 years	4	4
14 years	0.1	1
14 years	0.1	0.2

Pancreatic amylase was elevated in 19 patients (90.5%), and lipase levels were raised in 17 patients (81.0%). Autoimmune markers were positive in four patients (19.0%), with elevated immunoglobulin G4 levels. Genetic testing was performed in two patients (9.5%) and demonstrated pathogenic mutations (PRSS1 and SPINK1). Genetic testing was unavailable for the remaining 19 patients (90.5%); therefore, a genetic aetiology could not be assessed in the remaining subjects (Table 3).

**Table 3:** Laboratory parameters in paediatric patients with pancreatic disorders (n = 21)

Parameters	Findings	N (%)	Laboratory Value/Reference Range
Serum calcium level (mg/dL)	Elevated	18 (85.7%)	8.5-10.5 mg/dL
	Low	3 (14.3%)	-
Serum pancreatic amylase (U/L)	Normal	2 (9.5%)	0-160 U/L
	Raised	19 (90.5%)	-
Serum lipase level (U/L)	Normal	4 (19.0%)	30-110 U/L
	Raised	17 (81.0%)	-
Lipid profile	Normal	21 (100%)	TC <200 mg/dL, LDL <100 mg/dL, HDL >60 mg/dL, TG <150 mg/dL
	Dyslipidaemia	0	-
HbA1c	Normal	20 (95.1%)	<5.7%
	Raised	1 (4.7%)	-
Autoimmune profile (ANA, IgG4, Anti-LKM, ASMA)	Negative	17 (81.0%)	ANA <1:40, ASMA <1:20, Anti-LKM <1:20, IgG4 <135 mg/dL
	Positive	4 (19.0%)	IgG4 >135 mg/dL
Genetic testing (only two patients underwent genetic testing)	Normal	0 (0%)	-
	Abnormal	2 (100%)	PRSS1 and SPINK1

Abdominal ultrasound findings were abnormal in five patients (23.8%) who demonstrated an atrophic pancreas with thinning of parenchyma, dilated pancreatic duct, and pseudocyst. CT scan findings varied, with eight patients (38.1%) exhibiting a moderately dilated pancreatic duct, while minimal pancreatic duct dilation was observed in five patients (23.8%). MRCP findings revealed minimal prominence of the pancreatic duct in nine patients (42.9%) and dilated ducts in five patients (23.8%). ERCP was performed in selected patients, with five patients (23.8%) requiring stent placement due to ductal strictures (Table 4).

**Table 4:** Imaging parameters in paediatric patients with pancreatic disorders (n = 21)

Parameters	Findings	N (%)	Reference/Notes
Ultrasound	Normal	16 (76.1%)	-
	Atrophic pancreas with thinning of parenchyma and dilated duct	3 (14.2%)	-
	Pseudocysts/walled-off collection around necrotic tail of pancreas	2 (9.5%)	-
CT scan abdomen	Minimal pancreatic duct	5 (23.8%)	-
	Moderately dilated pancreatic duct	8 (38.1%)	-
	Swollen pancreas with main duct dilatation	2 (9.5%)	-
	Pancreatic atrophy	4 (19.0%)	-
	Retro-gastric pancreatic pseudocyst	2 (9.5%)	-
Endoscopic retrograde cholangiopancreatography	Normal	3 (14.3%)	-
	Stent placed	5 (23.8%)	Pancreatic duct stricture
Magnetic resonance cholangiopancreatography	Minimal prominence of pancreatic duct	9 (42.9%)	>2-2.5 mm in diameter
	Swollen pancreas	3 (14.3%)	-
	Atrophic pancreas	4 (19.0%)	-
	Dilated pancreatic duct	5 (23.8%)	>3-5 mm in diameter

Medical management was initiated in all patients and included analgesics (95.2%), dietary modification (85.7%), and pancreatic enzyme supplementation (52.4%). Surgical intervention was required in 11 patients (52.4%). Among those who underwent surgery, Frey’s procedure was the most commonly performed surgical intervention (38.0%). ERCP-guided therapeutic stent placement was performed in five patients (23.8%). Post-treatment outcomes were assessed via telephone follow-up at six weeks and three months. Sustained treatment success, defined as being symptom-free, was reported in 19 patients (90.5%), while two patients (9.5%) were lost to follow-up (Table 5).

**Table 5:** Treatment modalities and post-treatment outcomes in paediatric patients with pancreatic disorders (n = 21)

Treatment	n (%)
Medical management	
Analgesics	20 (95.2%)
Enzyme therapy	11 (52.4%)
Diet	18 (85.7%)
Naso-jejunal gastric tube	1 (19.0%)
Surgical management	
Nil	6 (28.6%)
ERCP guided stent placement	5 (23.8%)
Frey’s procedure	8 (38.0%)
Whipple procedure	1 (4.8%)
Puestow procedure	1 (4.8%)
Post treatment outcomes (at six weeks and three months)	
Lost to follow-up	2 (9.5%)
Sustained treatment success	19 (90.5%)

Among the study participants, complications observed were pancreatic calculi (19.0%), pseudocyst formation (9.5%), and diabetes mellitus (4.8%). Pancreatic insufficiency was assessed clinically based on a history of steatorrhoea, as the faecal elastase test was not feasible due to financial constraints (Table 6). 

**Table 6:** Complications in study population (N = 21)

Complication	N (%)
Pseudocyst	2 (9.5%)
Diabetes mellitus	1 (4.8%)
Pancreatic stones	4 (19.0%)
Pancreatic insufficiency	11 (52.4%)

## Discussion

CP was previously considered relatively uncommon in the paediatric population; however, increased awareness and improved diagnostic modalities have revealed a rising incidence among children worldwide [2]. Despite this, data from low- and middle-income countries remain limited.

A notable finding in our cohort was the high rate of parental consanguinity, suggesting a potential genetic predisposition and aligning with findings from prior regional studies [6]. The gene defects identified in our two patients, PRSS1 and SPINK, are among the most commonly implicated genetic factors in paediatric CP [7]. However, genetic studies could not be performed in most patients due to the lack of in-house genetic testing facilities at our centre. Although testing was outsourced, the high costs imposed a significant financial burden, limiting its widespread use. This underscores the critical importance of accessible and affordable genetic testing, as early identification of pathogenic mutations in patients and their parents can aid in confirming the diagnosis, guiding clinical management, enabling appropriate genetic counselling, and facilitating early screening of at-risk family members.

The aetiology of CP in children is diverse. In our study, the majority of cases were idiopathic, similar to findings reported by Gariepy et al., where a substantial proportion of paediatric cases did not have an identifiable cause [8]. Other aetiologies such as hyperlipidaemia, cystic fibrosis, and drug-induced pancreatitis were not identified in our cohort. Autoimmune pancreatitis is very rare in children; however, a small subset of patients in our study demonstrated elevated IgG4 levels that were not the cause of CP. These patients were managed conservatively with close multidisciplinary follow-up, consistent with existing literature that identifies IgG4 as a useful biomarker in selected cases [9].

Epigastric pain was the most common presenting symptom and significantly impacted quality of life, a finding consistent with previous longitudinal studies [10,11]. Elevated pancreatic enzymes were observed in most patients at presentation, reflecting acute exacerbations on a background of chronic disease. Stabilisation of enzyme levels following treatment and symptom resolution aligns with observations reported by Suzuki et al., supporting the concept of acute-on-CP in paediatric patients [12].

Radiological Imaging played a central role in diagnosis and disease characterisation. Abdominal ultrasound was adopted as the initial imaging modality due to its accessibility and safety profile; however, its diagnostic yield was limited, consistent with prior reports highlighting its operator dependence [13]. Cross-sectional imaging with abdominal CT scan provided improved anatomical detail, particularly in identifying ductal dilation and parenchymal changes associated with CP [14]. MRCP emerged as a valuable non-invasive modality, allowing detailed evaluation of pancreatic ductal morphology without radiation exposure. Our findings support existing evidence favouring MRCP over ERCP for diagnostic evaluation in paediatric patients [15,16].

ERCP was initially used as both a diagnostic and a therapeutic measure. It was reserved for therapeutic indications in our patients, particularly in patients with ductal strictures and stones. In our cohort, ERCP-guided stent placement provided symptom relief in selected cases consistent with Guo et al.’s study [17]. However, literature suggests that surgical intervention may provide long-term pain control compared to endoscopic therapy alone [18].

Medical management remains the first-line treatment for paediatric CP and focuses on pain control, nutritional optimisation, and pancreatic enzyme replacement. In our study, lifestyle modification and pharmacologic therapy resulted in symptomatic improvement in many patients along with a positive response to naso-jejunal gastric tube feed; however, a substantial proportion required surgical intervention due to refractory pain. Frey’s procedure was the most commonly performed surgery on patients who had atrophic changes with dilated pancreatic duct on their CT scan and MRCP, and was associated with favourable outcomes, consistent with published paediatric surgical series [19].

While none of the patients in our study underwent total pancreatectomy with autologous islet transplantation, current international recommendations support its use in selected children with hereditary pancreatitis and intractable pain to preserve endocrine function [20].

Complications observed in our study were pancreatic calculi, pseudocyst formation, pancreatic insufficiency, and diabetes mellitus. Pancreatic insufficiency was evaluated clinically, as faecal elastase testing could not be performed due to financial constraints. The single case of diabetes mellitus reflects the risk of endocrine insufficiency, though the low incidence may be influenced by the study’s limited sample size. All complications were managed appropriately according to standard clinical protocols. Clinical assessment of pancreatic exocrine function provides a practical approach in resource-limited settings [21].

Given the challenges of in-person follow-up due to the remote residence of many patients, follow-up was conducted via telephone, with a duration ranging from six weeks to three months. Post-surgical outcomes reported by parents indicated resolution of pain. This is consistent with the literature, which demonstrates that surgical intervention in similar cases is associated with favourable outcomes [22]. 

Overall, this study emphasises the importance of a multidisciplinary approach to paediatric CP, particularly in settings with limited diagnostic and therapeutic resources.

Limitations

This was a retrospective, single-centre study, with a small sample size, which may affect the findings. Genetic testing was unavailable for over 90% of patients, likely underestimating the contribution of inherited causes of CP in this study. Pancreatic exocrine insufficiency was assessed clinically rather than through faecal elastase testing because of financial limitations. Additionally, the short follow-up period of six weeks to three months communicated on the telephone may not fully reflect the extent of irreversible pancreatic injury, and the favourable surgical outcomes reported should be interpreted cautiously.

## Conclusions

Paediatric CP is an increasingly recognised condition that presents predominantly with recurrent abdominal pain and is frequently idiopathic in aetiology. Clinical assessment, biochemical investigations, and radiological advancement are crucial in diagnosing patients. Improved access to genetic testing and long-term follow-up may further enhance understanding of disease aetiology and outcomes in paediatric populations. In resource-limited settings, a practical, clinically driven diagnostic approach supported by multidisciplinary team collaboration can facilitate timely intervention and optimise patient care. Medical therapy remains the cornerstone of initial management, and surgical procedures were associated with favourable short-term outcomes in selected patients in our study.
